# The development of a home-based technology to improve gait in people with Parkinson's disease: a feasibility study

**DOI:** 10.1186/s12938-023-01066-2

**Published:** 2023-01-19

**Authors:** Galit Yogev-Seligmann, Naomi Josman, Noemi Bitterman, Sara Rosenblum, Sitar Naaman, Yafit Gilboa

**Affiliations:** 1 Department of Occupational Therapy, Faculty of Social Welfare & Health Sciences, University of Haifa, 3498838 Haifa, Israel; 2 Technion, Israel Institute of Technology, Haifa, Israel; 3 Department of Physical Therapy, Faculty of Social Welfare & Health Sciences, University of Haifa, Haifa, Israel; 4 School of Occupational Therapy, Faculty of Medicine, The Hebrew University of Jerusalem, Jerusalem, Israel

**Keywords:** Parkinson’s disease, Freezing of gait, Technology, External cues, Metronome, Light stripes, Smart home, Mobility, Patient satisfaction, Physiotherapy

## Abstract

**Background:**

People with Parkinson’s disease (PwP) may experience gait impairment and freezing of gait (FOG), a major cause of falls. External cueing, including visual (e.g., spaced lines on the floor) and auditory (e.g., rhythmic metronome beats) stimuli, are considered effective in alleviating mobility deficits and FOG. Currently, there is a need for a technology that delivers automatic, individually adjusted cues in the homes of PwP. The aims of this feasibility study were to describe the first step toward the development of a home-based technology that delivers external cues, test its effect on gait, and assess user experience.

**Methods:**

Iterative system development was performed by our multidisciplinary team. The system was designed to deliver visual and auditory cues: light stripes projected on the floor and metronome beats, separately. Initial testing was performed using the feedback of five healthy elderly individuals on the cues’ clarity (clear visibility of the light stripes and the sound of metronome beats) and discomfort experienced. A pilot study was subsequently conducted in the homes of 15 PwP with daily FOG. We measured participants' walking under three conditions: baseline (with no cues), walking with light stripes, and walking to metronome beats. Outcome measures included step length and step time. User experience was also captured in semi-structured interviews.

**Results:**

Repeated-measures ANOVA of gait assessment in PwP revealed that light stripes significantly improved step length (*p* = 0.009) and step time (*p* = 0.019) of PwP. No significant changes were measured in the metronome condition. PwP reported that both cueing modalities improved their gait, confidence, and stability. Most PwP did not report any discomfort in either modality and expressed a desire to have such a technology in their homes. The metronome was preferred by the majority of participants.

**Conclusions:**

This feasibility study demonstrated the usability and potential effect of a novel cueing technology on gait, and represents an important first step toward the development of a technology aimed to prevent FOG by delivering individually adjusted cues *automatically*. A further full-scale study is needed.

*Trial registration* This study was registered in ClinicalTrials.gov at 1/2/2022 NCT05211687.

**Supplementary Information:**

The online version contains supplementary material available at 10.1186/s12938-023-01066-2.

## Background

As their disease progresses, people with Parkinson’s disease (PwP) experience increasingly severe difficulties in mobility that negatively affect their function and quality of life (QoL) [1]. Alterations in gait are common and include reduced gait speed, arm swing, and stride length; and increased stride-to-stride variability which is a marker of a rhythmicity and automaticity related to gait unsteadiness and fall risk [2]. In addition, 20–60% of PwP experience freezing of gait (FOG), a sudden, brief inability to move forward, despite an intention to walk and is a major cause of falls [3, 4]. External cueing is a non-pharmacological approach used to mitigate gait disturbances and FOG among PwP. External cues include temporal or spatial stimuli provided (by someone or something else) to facilitate gait initiation and continuation [5, 6]. It is suggested that external sensory cueing can compensate for the defective rhythm generator of the basal ganglia [5]. The use of auditory, visual, or somatosensory cues can provide timing or spatial information to PwP for movement [7–9]. External cues facilitate gait by providing means to guide movement that rely on alternative motor pathways, such as the pre-motor area and the cerebellum [10, 11].

Cueing can be delivered by various modalities; the most common are visual (e.g., spaced lines on the floor) and auditory (e.g., rhythmic metronome beats) [5, 12]. Meta-analyses and systematic reviews [5, 12, 13] provide evidence of significant, immediate effects of cueing on gait disorders among PwP, including significant improvements in cadence, stride and step length, and gait speed [5, 12–14]. Most of the evidence about the effectiveness of cueing refers to gait impairments other than FOG, while information about the effect of cueing on FOG is relatively limited [8]. Spildooren et al. [15], for example, found that auditory cueing was effective in preventing FOG while turning. In another study, cueing training in the homes of PwP successfully reduced severity of freezing, although these effects were not sustained after cueing was removed [16]. In addition, a recent study showed that the effect of visual cueing on FOG diminished in the presence of anxiety that was provoked by walking in a threatening virtual environment [17].

Despite the proven benefit of cues, implementation is currently performed primarily using non-technological means, such as paper stripes glued to the floor, and, therefore, is rarely implemented at home. Although many technological methods have been suggested for the identification of FOG (see, e.g., [8, 18–23]), the technological means of delivering external cues that are available on today’s market are generally limited to walkers or canes that project red laser stripes on the floor and a smart-phone metronome application [24]. We postulate that a main problem with these solutions, however, is that they require manipulation of applications and may cause dependence on a mobility aid even among patients who walk independently. Previous studies proposed other technologies to facilitate external cues that require manipulation of equipment and need to be worn [25–29], such as wearable sensors, smartphones, earphones [6, 26, 30], Google glasses [27, 28], and the “laser-shoe” [25]. These proposed systems suffer from potential barriers that characterize wearable technologies, such as low compliance, forgetfulness, and loss of the wearable devices. Moreover, as far as we know, the clinical utility of these technologies has yet to be documented. Indeed, a 2021 survey conducted among 4987 PwP (42.8% with FOG) [31] found that of seven compensatory strategies presented, external cueing was the least frequently used. The authors suggest that PwP may find other compensatory strategies that do not require special devices (e.g., laser shoes, metronomes) or adaptations to the environment [31] more accessible and feasible.

To overcome these limitations, we developed a non-wearable technological solution that adjusts to the user’s baseline gait parameters and delivers external cues for PwP. We report here on the first step of development process and the initial pilot testing of a future novel technology designed to improve gait and prevent FOG among PwP, for use in the home. The specific aims of this feasibility study were to: (a) describe the iterative development process of the technology; (b) test a prototype of the technology on healthy older adults; (c) test the impact of the technology on gait among PwP in their homes. (d) obtain feedback on the user experience. First, the technology was developed (step 1: aims a and b), followed by a pilot test in the home environment (step 2: aims c and d).

## Methods

The study was approved by the Ethics Committee of the Faculty of Social Welfare & Health Sciences, University of Haifa (approval number 191/21). All participants signed an informed consent prior to their participation.

### Step 1: Iterative system development and testing by healthy older adults

**Fig. 1:**
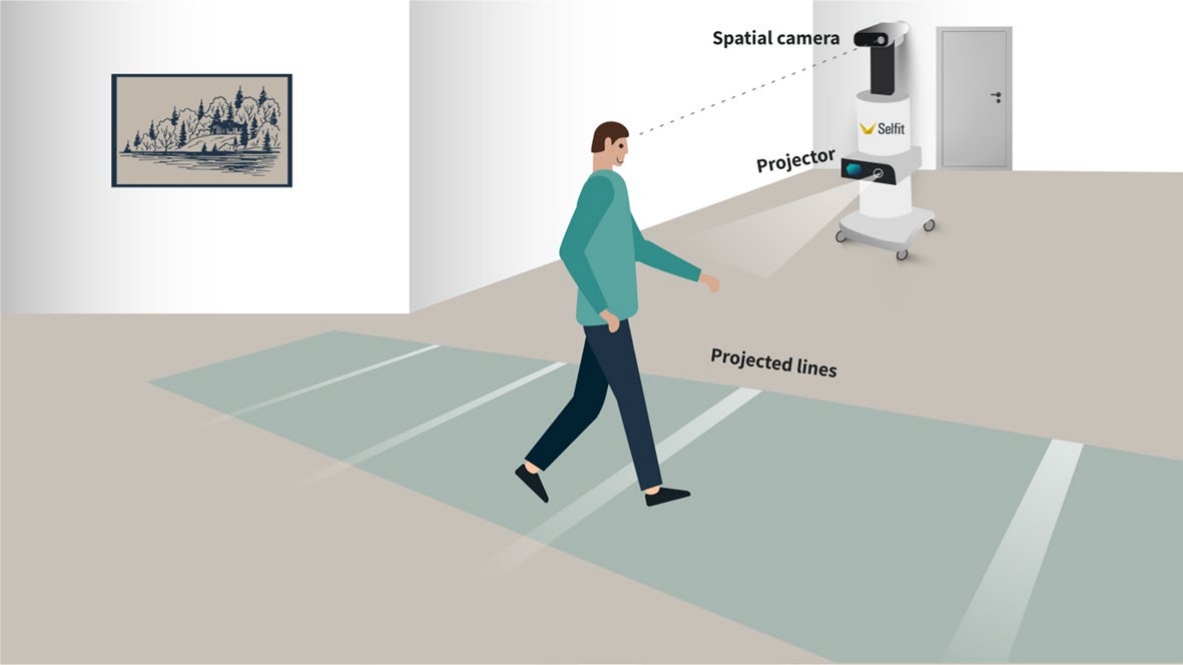
Illustration of the system. In the baseline condition, a spatial camera captures the user’s gait parameters, which are analyzed and used to determine cue frequency. The image illustrates the light stripes projected on the floor

A multidisciplinary team that included researchers with experience in Parkinson’s disease (Author GY-S), aging (Author NJ), rehabilitative technologies (Author YG), and smart home architecture (Author NB) designed and developed the system (illustrated in Fig. 1) in collaboration with Selfit Medical Ltd. (https://www.selfitmedical.com/). The system is designed to deliver external visual and auditory cues (i.e., projected light stripes and metronome beats, alternatively) that are personally adjusted to the user’s gait parameters, and records gait parameters during walking and attending to the external cues. In this stage of development, we first aimed to capture the experiences of PwP with FOG who use these external cues at home. Therefore, external cueing was activated by and in the presence of the research assistant.

### Hardware and software components of the technology

For each test, the following steps were performed using a Selfit Pro system, which comprises a computer, a projector and a spatial camera (MS Kinect Azure camera & Software Developer Kit; SDK): (a) The floor was detected and calibrated (internal to the SDK) and floor coordinates were generated; (b) The user was detected by Azure SDK, and the user’s skeletal joints were detected by the camera; (c) The position of each detected skeletal join was identified by Azure SDK; Software developed by Selfit Ltd calculated each foot position from the skeletal information. The system logged each leg interaction (standing in place) in relation to the floor coordinates. Steps were counted only if the counter leg was standing in place during movement; start and end-points were recorded. Steps were marked as completed only once the next step commenced.

**Fig. 2:**
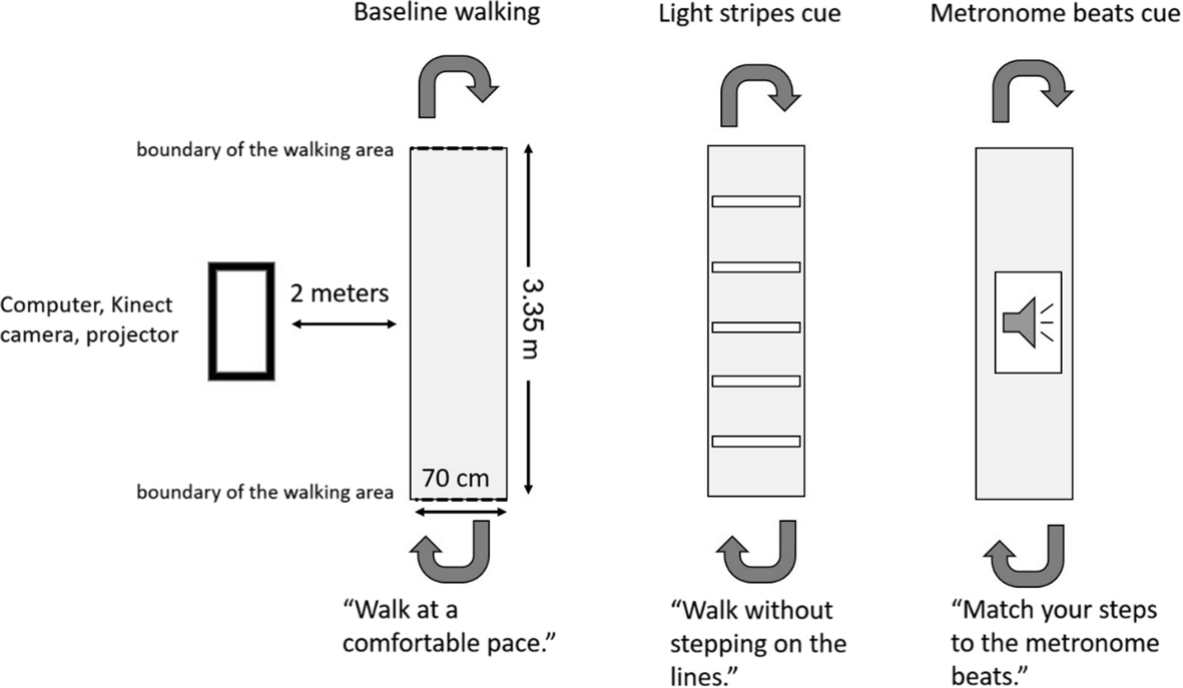
Illustration of the experimental setting. In each walking condition (i.e., baseline, light stripes, and metronome), participants walked back and forth nine times

The software checked and marked steps as not valid if: (a) Location coordinates of the steps were outside the detection boundaries (marked on the floor as the walking area; or (b) Positions of legs had abnormal coordinates (e.g., twisted or not aimed in the correct direction). The software stored step length, average leg height, cadence, and time for valid steps. Step length was calculated as the distance between the coordinates of the start and end-points and was averaged for the right foot step length and left foot step length. Step time was calculated as the difference between the start-time and end-time stamps. The software counted each time the individual crossed a boundary of the walking area (see Fig. 2). In each condition (baseline, light stripes, metronome beats), participants completed 9 passes of the walking area. Once the baseline condition was completed, the software calculated the user’s average valid step length, cadence, and step time. Step length and cadence were subsequently used to determine the distance between the light stripes and the frequency of the metronome beats in the cued walking conditions. The system projected light stripes on the floor according to the floor coordinates captured by the camera, and delivered metronome beats through the computer speaker. Kinect was previously reported to be a valid assessment tool for spatiotemporal gait parameters [32].

### Clinical considerations underlying system development

#### Cue modalities

Light stripes (visual cues) and metronome beats (auditory cues) were selected as the modalities to be delivered by the technology, as these are the most common types of cueing, and their positive effect has been demonstrated [5, 12, 13]. As each patient may have their own preferred cueing modality, we designed the technology to allow participants to experience both type of modalities (not simultaneously).

#### Cueing frequency

The distance between the light stripes was designed to be 110% of the user’s baseline step length [33] to encourage the user to take long steps. Beats frequency was calculated to 90% of the user’s cadence. This pace is recommended for PwP who experience FOG [33]. The number of light stripes was determined by the length of the walking area (⁓ 3.5 m) and by the distance between the lines calculated for each user according to their step length (on average, 4 or 5 stripes were projected on each walking area).

The system was initially tested on five healthy older adults with no neurological or orthopedic condition that potentially affects gait (age 64–74; four women and one man) who were recruited in a convenience sample. Healthy participants were tested in GYS’s lab in the University of Haifa and were asked to walk in the same experimental protocol that was later administered to the PwP with FOG participants (see step 3). Participants then completed a brief feedback questionnaire regarding the clarity of the light stripes and the sound of the metronome beats, and reported any discomfort they experienced during the activation of each cue.

### Step 2: Pilot testing in the home environment: assessing potential effects on gait and user experience

#### Participants

Participants were recruited from the community through an advertisement on the Israeli Parkinson Association website. PwP with FOG who were interested in participating were initially screened by telephone according to the following eligibility criteria: (a) able to walk independently; (b) reports daily episodes of FOG, as indicated by the response to item 2 of the New FOG Questionnaire (“How frequently do you experience freezing episodes?”) [34]; (c) score > 18 on the Telephone Version of the Mini-Mental State Examination (TMMSE) [35]; (d) intact hearing and vision by self-declaration; and (e) does not suffer from any neurological condition other than Parkinson’s disease.

#### Procedure

The study was conducted in one session lasting approximately 1.5 h in a wide room (usually the living room, minimum area of at least 4 × 3 m). Participants were asked in advance to take their medication as usual and the test was scheduled to ensure that participants were under the influence of the anti-parkinsonian medications at the time of the assessment (“ON state”).

##### Setting and experimental protocol

The system was placed at a distance of two m from the target walking area (see Fig. 2). In each of three walking conditions, participants were asked to walk to the end of the walking area and back, for a total of nine times. Turns were performed outside the walking area and were not recorded by the system. Walking conditions were counter-balanced between participants to reduces the risk of order bias. Each walking condition was completed within approximately 60 to 90 s.

1. Baseline walking condition (no cues): Participants walked at a comfortable pace. The cued walking area was a rectangle (70 cm × 3.35 m) marked by a white light projected on the floor. On the basis of the steps recorded in this walking condition, the software automatically calculated step length and cadence, and these were used to determine the parameters of the cueing modalities (i.e., the distance between the light stripes and the frequency of the metronome beats).
2. Visual cue walking condition (light stripes): The walking area was compromised of 4–5 white light stripes (70 cm × 5 cm, see Fig. 2; The number of stripes was determined automatically on the basis of the participant’s step length calculated in the baseline condition. The space between the stripes was 110% of the participant’s step length [33]. Participants were instructed to walk over the stripes without stepping on them.

3. Auditory cue walking condition (metronome beats): Metronome beats were delivered through the system. Beat frequency was calculated to 90% of the patient’s cadence. The walking area was compromised of an illuminated rectangle projected on the floor. Participants were instructed to match their steps to the rhythm of the metronome beats.

### Measures

#### Clinical characterization of the sample

(a) a demographic questionnaire developed specifically for this study; (b) Unified Parkinson’s Disease Rating Scale (UPDRS) [36], a four-part assessment tool for evaluating motor and non-motor aspects of daily living, behavior, mood, and complications in PwP. In this study we used Part 2 (Motor Aspects of Experiences of Daily Living, based on patient’s interview, maximum score 52) and Part 3 (Motor Exam, which is a physical examination of motor symptoms, maximum score 132); Higher scores on the UPDRS indicate greater disease severity; (c) Hoehn and Yahr’s staging of the disease [37] (d) New Freezing of Gait questionnaire, which is a ten-item scale that evaluates FOG severity (maximum score 32; Higher scores indicate increased severity of FOG, such as higher frequency and longer duration of FOG events)[34].

#### User experience

After completing the walking sessions in all three conditions, participants were asked to participate in a semi-structured feedback interview based on nine questions, including six multiple-choice questions (see Table 3), two open-ended questions, and two items that captured participants’ perceived magnitude of change in gait while walking with the light stripes and the metronome, using a global rating of change scales on a 10-point scale from -5 (“much worse gait”) to 5 (“much better gait”) [38].

#### Gait assessment

Participants’ step length (cm) and step time (sec) in each walking condition were calculated by the system and used to evaluate the effect of the cueing (visual and auditory) on gait. Mean values, standard deviations, and the coefficient of variation (SD divided by the mean × 100) were calculated for step length and step time in each walking condition.

### Statistical analysis

The system’s usability was analyzed with a combination of descriptive statistics and qualitative analysis of the feedback. Answers to open-ended questions were analyzed using qualitative content analysis [39]. Researchers compiled the responses to open-ended questions on an Excel spreadsheet, generated codes from the responses in open coding, and then developed categories and subcategories. Through discussion, the research team agreed on codes, categories, and subcategories. The interviews were conducted in Hebrew and direct quotes were translated into English by the authors for this manuscript.

### Gait analysis

The following gait parameters were included in the analysis: step length (cm), step time (sec), step length variability (%), step time variability (%). Variability measures were quantified using the coefficient of variation, e.g., step time variability = 100 (standard deviation/average step time). Outcome variables were tested for normality using the Shapiro–Wilks test*.* Gait variables followed a normal distribution and, therefore, were analyzed using parametric tests. Equal of variance was assumed for all parameters.

To examine the effect of the external cue conditions on gait, a repeated measures ANOVA was conducted, where baseline walking was compared to the external cue conditions. Effect sizes (partial eta-squared) were interpreted as small ≤ 0.01, moderate ≤ 0.07, and large ≤ 0.14 [40]. For significant main effects, a post-hoc analysis was performed using Bonferroni pairwise comparisons. The significance level was set at *p* < 0.05. Given the exploratory nature of the study, multiple testing corrections were not performed in the statistical analyses.

## Results

### Step 1: Iterative system development and testing by healthy older adults

In general, we were able to successfully administer the experimental protocol using the technology as planned, first in a group of five healthy adults that included four females (mean age 66.80 ± 4.14, years of education 18.40 ± 1.67) and then in a group of 15 PwP with FOG. No adverse events related to the operation of the system were reported.

### Step 2: Testing in home environment: pilot study on potential effect of the system on gait and user experience

A total of 31 PwP were recruited, but only 17 PwP with FOG were eligible to participate in the study. Two patients canceled their participation due to a COVID-19 outbreak. Participants’ characteristics are summarized in Table 1. Participants’ mean age was 64.66 ± 9.12 years (age range 49–77), and four participants (26.7%) were female. Telephone Version of the Mini-Mental State Examination (TMMSE) score was 24 ± 1 (range 23–26), which is comparable to an in-person Mini-Mental State Examination (MMSE) score of 25 [41], indicating that no participant suffered from suspected dementia. The mean number of falls experienced by participants in the past 12 months was 4.5 ± 7.99 (range 0–30). All participants reported that they had not previously used stripes or metronome beats as cueing modalities, although several participants reported experience with cognitive strategies (e.g., counting, using self-orders) or other common techniques to avoid FOG (e.g., kicking objects). According to Hoehn and Yahr’s staging, participants were in a mild stage of the disease. Overall, the scores in the NFOGQ were high. Individual participants’ characteristics are presented in Additional file 1.

**Table 1:** Demographic and clinical characteristics of the PwP sample (*N* = 15)

	Mean ± SD
Age (years)	64.67 ± 9.12
Sex	4 (26.7%) Females 11 (73.3%) Males
Education (years)	14.5 ± 2.3
Time since diagnosis (years)	14 ± 9
TMMSE	24.7 ± 1.1
UPDRS Part 2—Motor Aspects of Experiences of Daily Living (score range 0–52)	23 ± 9
UPDRS Part 3—Motor Examination (score range 0–132)	36 ± 11
NFOGQ (score range 0–32)	21 ± 5
Levodopa Equivalent Dose (mg)	1122 ± 690
Hoehn and Yahr stage	2.3 ± 0.5

UPDRS = Unified Parkinson’s disease rating scale, TMMSE = Mini-Mental state examination—telephone version

### Effect of external cues on gait

All participants completed the gait assessment protocol. No participant had FOG events while performing the gait protocol. Table 2 summarizes the effects of light stripes and metronome beats on step length, step time, and the variability (coefficient variation) of these parameters. Participants’ gait parameters at baseline were similar to previously reported values and are indicative of mid-stage disease [42]. All gait parameters were normally distributed based on the Shapiro–Wilk test. The ANOVA revealed that light stripes, but not metronome beats, significantly increased step length and step time. However, light stripes significantly increased the variability of gait parameters in comparison with baseline walking. Individual participants’ gait measures are presented in Additional file 2.

**Table 2:** Effect of external cues on gait parameters (*N* = 15)

	Baseline Mean ± SD	Light stripes Mean ± SD	Metronome beats Mean ± SD	Main effect *F*_(2,28)_, p partial eta^2^ (ES)	Bonferroni post-hoc
Step length (m)	0.48 ± 0.06	0.53 ± 0.10	0.48 ± 0.08	6.17, 0.006 0.31	Baseline < light stripes (*p* = 0.009)
Step time (sec)	0.61 ± 0.05	0.68 ± 0.08	0.62 ± 0.05	8.75, 0.001 0.38	Baseline < light stripes (*p* = 0.030) Metronome < light stripes (*p* = 0.019)
Step length variability (%)	19.54 ± 6.54	24.74 ± 6.67	20.52 ± 8.71	4.64, 0.018 0.25	Baseline < light stripes (*p* = 0.041)
Step time variability (%)	17.31 ± 3.72	21.08 ± 7.20	18.93 ± 7.36	4.03, 0.029 0.22	Baseline < light stripes (*p* = 0.048)

### User experience

Table 3 summarizes participants’ feedback about walking with external cues, based on their responses to the six multiple-choice items that addressed cue clarity, discomfort, perceived change in walking, possible effectiveness in preventing FOG, desire to have such system at home, and preference of cues. Overall, participants were satisfied with the cues provided by the system. The cues were clear to the participants. Most participants (*n* = 13, 87%) felt that both cueing modalities positively affected their gait, and would like to have such technology at home. The two participants who did not wish to have the technology at home stated that they were not sure whether the technology would help them and that they were in greater need of a technology to improve outdoor walking. The preferred cueing modality is metronome beats. Two participants reported some discomfort with the weak sound while using the cues (see details below).

**Table 3:** Distribution of the PwP sample responses to the multiple-choice items in the semi-structured feedback interview (*N* = 15)

	Light stripes			Metronome beats		
	Yes	No	Maybe	Yes	No	Maybe
1. Did you see the light stripes/ hear the metronome beats clearly?	15	0	0	15	0	0
2. Did you experience any discomfort while walking over the stripes/ using the metronome beats?	2	13	0	2	13	0
3. Did you feel any change in your walking while using the light stripes/ metronome beats?	13	2				
4. Do you think that the light stripes might be effective for preventing FOG?	6	3	6	8	0	7
5. Would you like to have such a technology installed in your home?	Yes—13; No—2; Maybe—0					
6. Which kind of cues would you prefer?	Metronome—10; Light stripes—3; Both—2					

In most cases, the subjective perception of change in gait and the objective change measured were consistent. In some participants, however, there was a discrepancy. For example, using external cues increased step length (compared to baseline) in two participants (#1,10) who reported no change in their gait. Several participants (e.g., #2,4,5) who felt walking with external cues changed their gait significantly, although no objective changes in their gait were recorded.

In addition to the multiple-choice questions, participants were asked to describe in their own words how the delivered cues affected their gait. This question was posed once with respect to the light stripes and once with respect to the metronome. Manual coding of interview responses to walking with light stripes revealed three main themes: sense of confidence, changes in gait, and need for concentration. The same three themes emerged from participants’ feedback regarding the metronome beats. Most participants stated that the cues increased their confidence in walking and had a positive effect on their gait. Several participants reported that walking with the cues required them to invest more effort to concentrate on walking. Examples of participants’ responses are detailed in Additional file 3.

### Perceived magnitude of change in gait while using external cues

**Fig. 3:**
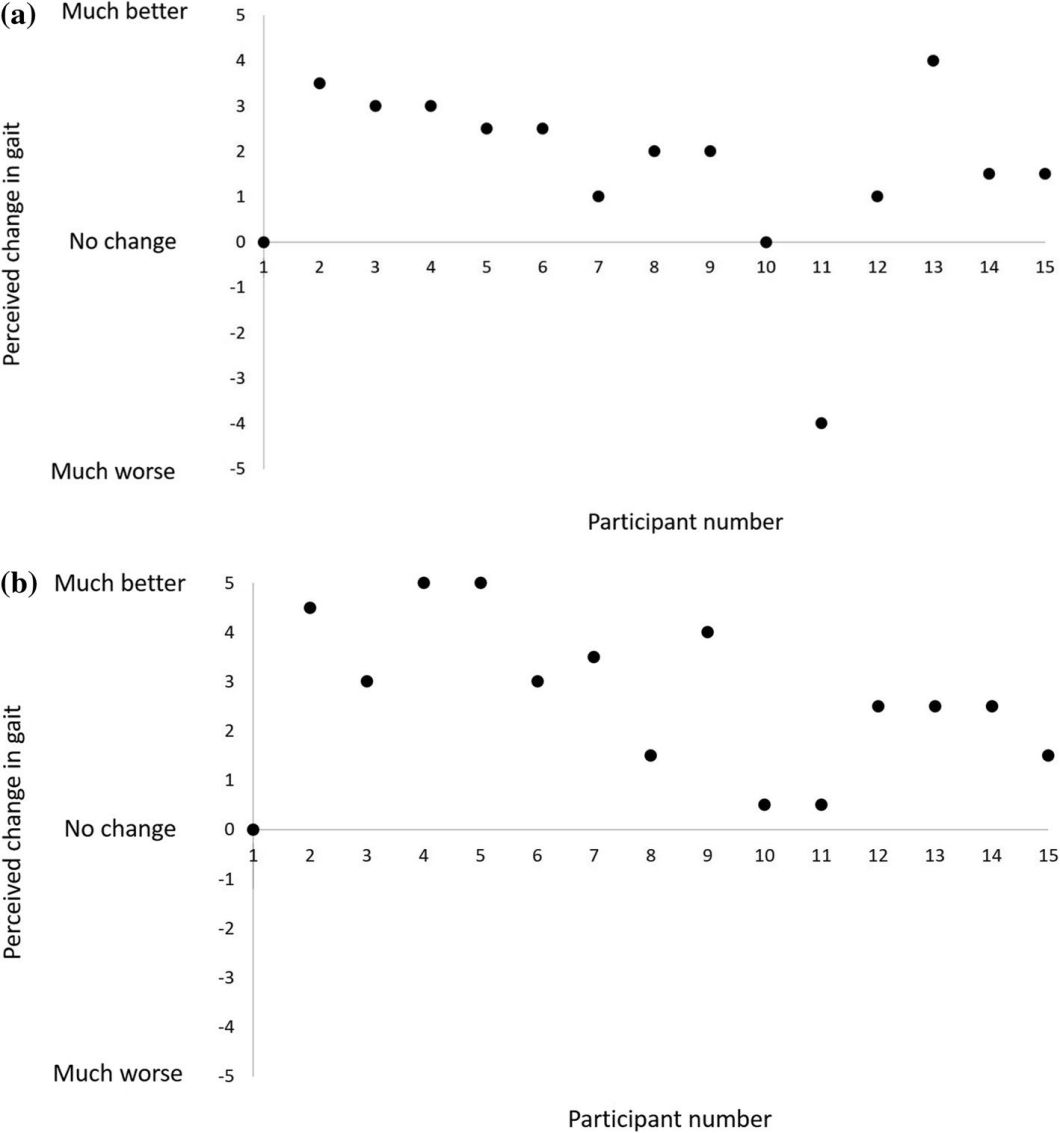
**a** Perceived magnitude of change in gait while using light stripes. **b** Perceived magnitude of change in gait while using metronome beats

Figure 3 summarizes the magnitude of change reported by participants following the use of the light stripes (Fig. 3A) and the metronome beats (Fig. 3B). The majority of participants (*n* = 12, 80%) reported that the visual cues led to a positive change, two participants (13%) reported no change in their gait, and one participant (7%) felt that the light stripes adversely affecting their gait. Similarly, the majority of participants (*n* = 14, 93%) reported a positive change following the use of the auditory cues, and one participant (7%) reported that the metronome beats did not change their gait.

## Discussion

This study demonstrated the usability of a technology for delivering individually adjusted visual and auditory cues in the homes of PwP with FOG. Gait assessment revealed that light stripes, but not metronome beats, significantly improved step length and increased step time. However, in contrast to these gait measures, increased variability in step time and step length was observed in the light stripes condition. Findings showed that most participants felt that both cues positively affected their gait and caused no discomfort, and stated that they would like to have such a technology installed in their home.

Results of the ANOVA model showed significant beneficial effects of light stripes on step length, but also showed an increase in gait variability in step length and time, in comparison with baseline walking. Walking in the metronome condition had no effect on gait parameters compared to baseline walking. Our results are consistent with the findings of Lee et al. [43], which showed that PwP with FOG benefited more from visual cues than from auditory cues. In addition, a previous review [6] suggested that although both types of modalities are useful for reducing FOG, visual modalities had a more powerful effect on gait in freezers and reduced FOG to a greater extent, compared to auditory modalities. It should be noted that the absence of a metronome effect on gait variables in the current study is contrary to previous studies and reviews that suggested that auditory cues increase gait speed and stride length [5, 12, 13] and reduce gait variability [44–46]. However, several studies reported that auditory cueing did not affect these parameters [47–49]. The increase in variability when the light stripes were used was previously explained by the different nature of the cues: Visual cues, which are spatial in nature, mainly seem to influence scaling and amplitude generation during walking, while temporal auditory cues tend to affect the timing of gait and inter-limb coordination [5, 6]. Therefore, the metronome beats (and not the light stripes) were expected to reduce variability in comparison with baseline walking.

Most participants perceived both cueing modalities as helpful, and noted that both modalities improved their gait, confidence, and stability. Only one-half of the participants, however, thought that cueing would be useful to mitigate FOG, while the remaining participants were uncertain, and three responded that the light stripes might not be useful. Participants’ uncertainty may be explained by the fact that the participants in this study had not previously experienced cueing modalities of stripes or metronome beats, and the single experience in this pilot study was an insufficient basis on which to develop an opinion on the usefulness of these cueing modalities for reducing FOG.

In this pilot study, 10 of the 15 participants said that they prefer the metronome, three participants preferred the light stripes, and two participants stated that they would like to have both (not simultaneously). This finding was surprising considering the fact that gait was objectively improved only by the light stripes. Furthermore, two participants reported that the light stripes had no effect on their gait (Fig. 2a), despite findings that showed that their step length increased by 17% and 18%, respectively, in this cueing modality. The paradox of user preference vs. performance has been documented [50–52]. Research in human factors indicates discrepancies between what users want and what is needed to improve their performance [50–52]. Andre and Wickens [51] suggested that preference may be affected by aesthetics, novelty, familiarity, or low effort. In accordance with that suggestion, that auditory beats may be more intuitive and familiar to most people (“it is like music” as noted by one of the participants) than lines on the floor, and, therefore, were preferred by most participants.

Finally, several participants reported that the metronome, but not the light stripes, assisted during turns. Although gait during turning was not measured in this feasibility study, metronome beats were audible even when participants stepped outside the walking area to turn. In contrast, light stripes were available as cues only within the boundaries of the walking area. Furthermore, the metronome modality does not require the user to look at the floor, thus may be enable more natural, safe walking.

The fact that no participant experienced FOG events, while completing the gait assessment protocol is a phenomenon previously described by others [53, 54], who reported that PwP who commonly suffer of FOG in daily living do not manifest FOG when under examination in a research or clinical setting. It is suggested that a conscious focus on gait, as commonly happens when PwP are under examination, results in better gait performance and reduced FOG manifestation [54]. This effect indicates that future assessment of the technology should be based on more extended patient experiences of the technology’s usefulness for mitigating FOG, to capture FOG events occurring in natural daily live settings when patients are not being observed by an examiner.

The next step of development aims to design a minimized, clinically useful home-based tool that automatically delivers cues (triggered by a proximity sensor) and assesses gait parameters at regular intervals for the optimal adjustment of cues. Although the system will not be designed to detect FOG events, it will be installed in locations, where patients report frequently FOG experiences FOG (e.g., corridors, kitchen, entrance to bathroom) and deliver cues whenever the user’s approach is detected.

The protype developed and tested in this study is the first step in the development of a future minimized technology that automatically delivers cues in locations in the home were PwP report frequently FOG episodes. Such technology may have the advantage of releasing patients from the need to wear or manipulate devices, as in previously suggested technologies, such as cell phone or glasses. Forgetfulness, apathy and motor difficulties contribute to PD patients not using cueing modalities, hence automaticity of delivery could improve the usability of cueing.

This study has several limitations. First, the sample was small and homogenous in terms of cognitive function, and the interpretation of the results should, therefore, be made with caution. Second, the dimensions of the protype precluded placement in cluttered or narrow spaces, where FOG commonly occurs. This may be one of the reasons that FOG were not detected among participants in the current pilot study. In the next step of development, a minimized setup will enable the installation of technology in small spaces, and hopefully enable a more direct measurement of the technology’s effect on FOG. Third, inclusion of a FOG provoking test in the study protocol might have assisted in the observation and measurement of the effect of cueing on FOG, and will be included in the future studies. Additional limitations concern the relatively short walking area used to measure gait, which may have influenced the magnitude of gait variability values. Although such path length was previously used by others who assessed the effect of cueing on gait in PwP [46, 47]. Further research should also include a training session for the use of external cues, since familiarity with the task may also affect gait variables.

Other limitations are the participants’ single experience in the use of the cues modalities as well as assessing the participants in an “ON” state, which limited the possibility to ecologically assess the usefulness of the technology in mitigating FOG. Moreover, the fact that no participants experienced FOG during the pilot test (possibly due to the examiner’s presence) limited the ability to assess the effect of the technology on FOG reduction. Finally, the Kinect has been validated mostly in healthy younger adults and only limited evidence of its validity in PwP is available [32, 55–57].

## Conclusions

The current feasibility study responds to the need for a technology that delivers cues in PwP’s homes. This study demonstrated the usability of a technological prototype and its positive effect on gait and potential effect on FOG. This is the first step toward the development of a home-based technology aimed to deliver individually adjusted cues automatically. The technology should, beyond the delivery of cues, plan to overcome other features of the disease that may affect its use.

## Supplementary Information

**Additional file 1: Table S1.** Individual participants’ characteristics.

**Additional file 2: Table S2.** Individual participants’ gait measures (average (SD)).

**Additional file 3: Table S3.** A summary of the responses received by the participants.

## Data Availability

The data sets used and/or analyzed in the current study are available from the corresponding author on reasonable request.

## Abbreviations

PwP: People with Parkinson’s disease
FOG: Freezing of gait
QoL: Quality of life
PD: Parkinson’s disease
SDK: Software Developer Kit
TMMSE: Telephone Version of the Mini-Mental State Examination
UPDRS: Unified Parkinson’s Disease Rating Scale
