# Supplementary material for: The development of a home-based technology to improve gait in people with Parkinson's disease: a feasibility study
Source: Biomed Eng Online. 2023 Jan 19;22:2. doi: 10.1186/s12938-023-01066-2 (PMC9851591; doi:10.1186/s12938-023-01066-2)
Supplement: Supplementary file 1 — Additional file 1: Table S1. Individual participants’ characteristics. [file 12938_2023_1066_MOESM1_ESM.docx]

| participant | Age (years) | Gender | Education  (years) | Time since diagnosis  (years) | UPDRS part2 | UPDRS  Part 3 | FOG-questionnaire | Levodopa Equivalent Dose (mg) | Hoehn and Yahr stage |
| --- | --- | --- | --- | --- | --- | --- | --- | --- | --- |
| # 1 | 71 | male | 12 | 26 | 18 | 21 | 27 | 865 | 2 |
| # 2 | 50 | male | 17 | 17 | 20 | 36 | 24 | 575 | 2 |
| # 3 | 72 | male | 17 | 18 | 25 | 36 | 14 | 1206 | 2 |
| # 4 | 49 | female | 12 | 19 | 19 | 23 | 25 | 2800 | 3 |
| # 5 | 61 | female | 16 | 13 | 32 | 51 | 26 | 801 | 3 |
| # 6 | 52 | male | 18 | 9 | 16 | 26 | 21 | 825 | 2 |
| # 7 | 69 | male | 13 | 17 | 37 | 36 | 28 | 1688 | 2 |
| # 8 | 71 | male | 12 | 37 | 41 | 50 | 26 | 2922 | 3 |
| # 9 | 62 | female | 13 | 6 | 14 | 31 | 21 | 760 | 2 |
| # 10 | 76 | male | 16 | 8 | 9 | 18 | 24 | 1518 | 2 |
| # 11 | 77 | male | 13 | 9 | 20 | 51 | 18 | 1175 | 3 |
| # 12 | 62 | male | 13 | 6 | 18 | 33 | 7 | 675 | 2 |
| # 13 | 61 | male | 17 | 3 | 17 | 39 | 19 | 300 | 2 |
| # 14 | 73 | male | 16 | 6 | 29 | 47 | 17 | 525 | 2 |
| # 15 | 64 | female | 12 | 12 | 35 | 49 | 26 | 825 | 3 |

Additional data

Table S1: Individual participants’ characteristics
