# Supplementary material for: The development of a home-based technology to improve gait in people with Parkinson's disease: a feasibility study
Source: Biomed Eng Online. 2023 Jan 19;22:2. doi: 10.1186/s12938-023-01066-2 (PMC9851591; doi:10.1186/s12938-023-01066-2)
Supplement: Supplementary file 2 — Additional file 2: Table S2. Individual participants’ gait measures (average (SD)). [file 12938_2023_1066_MOESM2_ESM.docx]

Additional data

Table S2: Individual participants’ gait measures (average (SD))

| participant | Step  length Base line (m) | Step length light stripes (m) | Steplength metronome  (m) | step time Base line  (sec) | Step time light stripes  (sec) | step time metronome  (sec) | Step length variability Base lineA  (%) | Step length variability light stripes (%) | Step length variability metronome (%) | Step time variability Base line (%) | Step time variability light stripes (%) | Step time variability metronome (%) |
| --- | --- | --- | --- | --- | --- | --- | --- | --- | --- | --- | --- | --- |
| # 1 | 0.63  (0.09) | 0.74  (0.13) | 0.68  (0.07) | 0.55  (0.08) | 0.54  (0.09) | 0.53  (0.06) | 14.40 | 17.57 | 10.65 | 14.33 | 15.73 | 11.90 |
| # 2 | 0.46  (0.11) | 0.41  (0.14) | 0.53  (0.11) | 0.63  (0.10) | 0.69  (0.15) | 0.62  (0.08) | 25.02 | 34.82 | 20.49 | 15.39 | 22.53 | 13.01 |
| # 3 | 0.45  (0.11) | 0.46  (0.15) | 0.43  (0.13) | 0.63  (0.13) | 0.63  (0.13) | 0.63  (0.13) | 24.85 | 33.48 | 29.47 | 20.88 | 20.05 | 19.86 |
| # 4 | 0.52  (0.04) | 0.60  (0.14) | 0.52  (0.04) | 0.58  (0.08) | 0.63  (0.11) | 0.59  (0.10) | 7.38 | 24.53 | 7.96 | 14.50 | 18.16 | 17.37 |
| # 5 | 0.40  (0.07) | 0.46  (0.10) | 0.40  (0.04) | 0.58  (0.10) | 0.66  (0.12) | 0.58  (0.07) | 18.23 | 21.85 | 11.89 | 17.14 | 18.26 | 11.58 |
| # 6 | 0.48  (0.08) | 0.54  (0.11) | 0.51  (0.12) | 0.67  (0.11) | 0.66  (0.18) | 0.65  (0.13) | 17.43 | 20.82 | 23.65 | 15.80 | 26.96 | 20.28 |
| # 7 | 0.49  (0.09) | 0.51 (0.14) | 0.49  (0.10) | 0.60  (0.13) | 0.59  (0.09) | 0.60  (0.10) | 17.55 | 28.46 | 20.79 | 21.49 | 15.48 | 16.91 |
| # 8 | 0.43  (0.13) | 0.46  (0.12) | 0.39  (0.13) | 0.68  (0.17) | 0.83  (0.34) | 0.72  (0.29) | 30.39 | 27.13 | 35.83 | 25.71 | 40.92 | 40.19 |
| # 9 | 0.39  (0.10) | 0.41  (0.13) | 0.46  (0.10) | 0.58  (0.12) | 0.74  (0.23) | 0.66  (0.18) | 26.16 | 31.82 | 22.30 | 20.48 | 30.83 | 26.68 |
| # 10 | 0.57  (0.09) | 0.68  (0.11) | 0.54  (0.09) | 0.61  (0.10) | 0.61  (0.12) | 0.63  (0.12) | 16.13 | 17.29 | 17.53 | 15.69 | 19.51 | 19.38 |
| # 11 | 0.40  (0.07) | 0.45  (0.14) | 0.41  (0.08) | 0.66  (0.14) | 0.85  (0.20) | 0.65  (0.09) | 19.47 | 31.23 | 20.12 | 20.66 | 23.61 | 14.66 |
| # 12 | 0.54  (0.11) | 0.60  (0.11) | 0.58  (0.11) | 0.77  (0.11) | 0.70  (0.10) | 0.72  (0.09) | 20.37 | 18.33 | 18.97 | 14.41 | 13.94 | 12.67 |
| # 13 | 0.50  (0.09) | 0.62  (0.07) | 0.52  (0.07) | 0.58  (0.07) | 0.65  (0.09) | 0.62  (0.09) | 17.20 | 11.95 | 13.61 | 12.11 | 14.30 | 15.03 |
| # 14 | 0.47  (0.13) | 0.47  (0.12) | 0.34  (0.12) | 0.60  (0.08) | 0.67  (0.12) | 0.57  (0.12) | 29.18 | 25.14 | 36.06 | 13.99 | 18.11 | 20.76 |
| # 15 | 0.45  (0.04) | 0.47  (0.12) | 0.39  (0.06) | 0.55  (0.09) | 0.77  (0.14) | 0.57  (0.14) | 10.05 | 26.03 | 14.98 | 17.07 | 17.86 | 23.69 |
