# Supplementary material for: The development of a home-based technology to improve gait in people with Parkinson's disease: a feasibility study
Source: Biomed Eng Online. 2023 Jan 19;22:2. doi: 10.1186/s12938-023-01066-2 (PMC9851591; doi:10.1186/s12938-023-01066-2)
Supplement: Supplementary file 3 — Additional file 3: Table S3. A summary of the responses received by the participants. [file 12938_2023_1066_MOESM3_ESM.docx]

Additional file 3

Below we summarize some of the responses received (#number of participant).

*Sense of confidence*

Participants described that the visual cues increased their confident in walking, e.g.: “I felt more confident in walking” (#4). Another participant described how the lines “allow me to plan the step, (enable) more awareness of the steps, and a sense of confidence, but it did not help in turning” (#2); “[gave me] a sense of frame and security” (#6). In addition, participants reported an increased sense of confidence using the metronome beats., for example: “feel safer” (#3), “I felt steady” (#11).

*Changes in gait:* Participants described the effect of the visual cues on their gait. For example, “[the light stripes] increase the step (length)” (#3), “[I was] lifting my legs up higher,” (#4)

“…a feeling that walking can and should be precise, feels familiar and correct, arranged the walk for me” (#6) “I was not dragging my legs” (#9). With respect to the effect of the metronome beats on gait, and emphasis on turning, participants noted, for example, “Adjusting the steps to the metronome made it easier to walk, also during the turns” (#2), “Walking is orderly” (#3), “It was fun to walk in sync with the ticking, joy, like music, helped also while turning” (#5), “Walking became a pattern, the speed was comfortable” (#11).

*Need for concentration:*

Several participants reported that walking with the light stripes required them to invest more effort to concentrate on the walk. For example,” “It was harder, I concentrated more on walking, my head was (looking) down” (#5), “Maintaining larger steps was inconvenient” (#7), “I needed a lot of concentration” (#9). On the need for concentration, several participants reported that the use of metronome was difficult: For example, “The metronome influenced (my walking), but I am not sure that it was for the better, it required an effort to match the pace of the metronome” (#8); “It requires practice and release to walk with the sound” (#6).
